# Improving TRAIL-induced apoptosis in cancers by interfering with histone modifications

**DOI:** 10.20517/cdr.2020.58

**Published:** 2020-10-09

**Authors:** Bao-Jie Zhang, Deng Chen, Frank J. Dekker, Wim J. Quax

**Affiliations:** University of Groningen, Department of Chemical and Pharmaceutical Biology, Groningen Research Institute of Pharmacy, University of Groningen, Groningen 9713 AV, The Netherlands.

**Keywords:** Epigenetics, histone modification, tumor necrosis factor-related apoptosis-inducing ligand, selective epigenetic inhibitors, apoptosis

## Abstract

Epigenetic regulation refers to alterations to the chromatin template that collectively establish differential patterns of gene transcription. Post-translational modifications of the histones play a key role in epigenetic regulation of gene transcription. In this review, we provide an overview of recent studies on the role of histone modifications in carcinogenesis. Since tumour-selective ligands such as tumor necrosis factor-related apoptosis-inducing ligand (TRAIL) are well-considered as promising anti-tumour therapies, we summarise strategies for improving TRAIL sensitivity by inhibiting aberrant histone modifications in cancers. In this perspective we also discuss new epigenetic drug targets for enhancing TRAIL-mediated apoptosis.

## Introduction

In humans, the genetic information (DNA) is contained in 23 chromosome pairs. These chromosomes are composed of DNA and histone proteins that form highly condensed chromatin. In parallel to genetics, the term “epigenetics” was originally defined to describe heritable changes that are not encoded in the DNA. Currently, epigenetics is used as a common term to describe chromatin modifications that regulate DNA-based processes including heritable and non-heritable changes^[1]^. The main players in epigenetic regulations are DNA modifications, histone modifications and non-coding RNAs. Histone modifications regulate, among other things, chromatin remodelling, which is closely related to regulation of gene transcription. For example, heterochromatin is usually tightly packed and prohibits gene transcription, while euchromatin is usually loosely packed and enables gene transcription^[2]^. Since epigenetics plays a crucial role in DNA-based processes, histone modifications are very important in cell growth in normal and disease states such as carcinogenesis.

Among various strategies to treat cancers, the selective induction of cellular apoptosis in cancer cells is considered as a promising therapeutic strategy. A well-known ligand to induce apoptosis is tumor necrosis factor (TNF)-related apoptosis-inducing ligand (TRAIL). Dulanermin is a TRAIL-based therapeutic containing amino acids 114-281 of human TRAIL, which has been developed as a clinical anti-cancer drug. An early phase I clinical study showed that dulanermin was well-tolerated by patients with advanced cancer. However, only 3% of the patients in this study responded to dulanermin treatment for a period longer than 6 months^[3]^. This may be due to TRAIL-resistance, which occurs in various type of cancer cells. TRAIL-resistance can be attributed to impaired TRAIL binding to death receptors, modified levels of apoptosis-related proteins, and reduced caspase functions^[4]^. In spite of this, TRAIL-based therapeutics are currently under clinical investigation. For instance, a phase I clinical study is recruiting participants to study the application of the novel TRAIL trimer SCB-313 for the treatment of malignant pleural effusions and peritoneal malignancies (NCT03869697 and NCT03443674). Moreover, phase I/II clinical studies with lung cancer patients using TRAIL expressed by mesenchymal stem cells are ongoing (NCT03298763). This demonstrates an active interest in the clinical application of TRAIL-based therapeutics.

In this review, we provide an overview of post-translational modifications of histones and the enzymes involved in the addition or removal of these modifications. We discuss small molecules targeting these enzymes and their anti-tumour effects. We connect this to targets involved in apoptosis as potential approach in cancer therapy. Finally, we summarize the current understanding of epigenetic mechanisms involved in sensitivity to TRAIL-induced apoptosis.

## Histone modifications

Histones are the central components of nucleosomes, in which a DNA string wraps around an octamer containing two copies of four core histones (H3, H4, H2A and H2B). These nucleosomes are organized like “beads” on DNA strings and are connected by histone protein H1 and further compacted to 30 nm-chromatin fibres, which are eventually condensed to form a chromosome. Therefore, histones provide structural support for chromosomes to provide organized packing of the DNA inside the nucleus. Unstructured histone tails are excluded from nucleosome cores and these tails are rich in lysine and arginine residues. Lysine residues are positively charged and provide charge-charge interactions with the negatively charged DNA, thus compacting the chromatin structure. Post-translational modifications occur mostly on the *N*-terminal tails of histones. These modifications play versatile roles in regulation of the structure and accessibility of the chromatin for transcription factors [Table 1].

**Table 1:** Histone modifications

Amino acids	Modifications	Positions	Nomenclature	Ref.
Arginine	Methylation	*H3R2/R8/R17/R26, H4R3, H2AR3	R-me1, R-me2s, R-me2a	[5,6]
	Citullination	*H3R2/R8/R17/R26/R42, H4R3, H2A, and H1	R-citrulline	[7]
Lysine	Methylation	*H3K9/K4/K36/K79/K27, H4K5/K20	K-me1, K-me2, K-me3	[8]
	Acetylation	*H3K9/K14/K56, H4K5/K12/K16	K-acetyl	[9,10]
	Propionylation	*H3K14	K-propionyl	[11]
	Butyrylation	*H3K14, H4K5/K8	K-butyryl	[11,12]
	2-hydroxyisobutyrylation	H2AK5/9/36/74/75/95/118, H2BK5/12/20/23/24/34/43/46/57/85/108/116/120, H3K4/9/14/18/23/27/36/56/64/79/122 H4K5/8/12/16/31/44/59/77/79/91	K-2-hydroxyisobutyryl	[13]
	Malonylation	*H2AK119	K-malonyl	[14]
	Succinylation	*H3K79	K-succinyl	[15]
	Crotonylation	H2AK36/118/119/125, H2BK5/11/12/15/16/20/23/34 H3K4/9/18/23/27/56, H4K5/8/12/16	K-crotonyl	[16]

*Specific positions which were identified to have certain effects in nuclear processes

### Modifications of arginine

#### Arginine methylation

Biologically, arginine methylation refers to a reaction in which a methyl group is transferred from *S*-adenosyl-*L*-methionine (SAM) to one or both omega nitrogens of an arginine amino acid residue. This transfer leads to formation of monomethylarginine (MMA), asymmetric dimethylarginine (ADMA) and/or symmetric dimethylarginine (SDMA). This methylation reaction is catalysed by N-arginine methyltransferases (PRMTs). All of the PRMTs can catalyse monomethylation of arginine to provide MMA. Type I PRMTs, including PRMT1, 2, 3, 4, 6 and 8, methylate MMA further to provide ADMA. Type II PRMTs, including PRMT5 and 9, methylate MMA further to provide SDMA. PRMT7 is classified as a type III enzyme that catalyses methylation of various substrates. Histone arginine methylation is directly associated with gene transcription. For instance, methylation at H3R2 blocks the ability to methylate H3K4, which is responsible for recruiting chromatin-remodelling enzymes to maintain a transcriptionally active state^[17]^. H4R3 has been identified as a binder of DNA methyltransferase DNMT3A^[18]^.

In contrast to arginine methylation, it is less clear which enzymes catalyse arginine demethylation. JMJD6 was initially reported to demethylate H3R2 and H4R3^[19]^, however this was disputed in later studies^[20-22]^. Recently, a new study reported that JMJD1B, a lysine demethylase, also demethylates arginine at H4R3^[23]^.

#### Arginine citrullination

A recently identified arginine post-translational modification is citrullination. This post-translational modification was already found in dozens of proteins, such as proteases, metabolic enzymes, and histones. The citrullination of histones is well-known to be involved in the formation of neutrophil extracellular traps (NETs), which is connected to innate immunity. In the process of clearing bacteria, the neutrophils secrete DNA, histones, and intracellular proteins to the extracellular space where they form NETs^[24]^. In comparison to the involvement of histone citrullination in immune response, the exact biological significance of histone citrullination in carcinogenesis is largely unclear^[7]^.

### Modifications of lysine

#### Lysine methylation

Lysine methylation is tightly regulated by “writers” (KMTs, methyltransferases) and “erasers” (KDMs, demethylases). Similar to PRMTs, KMTs also employ SAM as co-factor to transfer one, two, or three methyl groups to specific histone lysine residues. More than 50 human KMTs and 30 KDMs have been identified^[25]^. Instead of global regulation of gene expression across different types of cells, KMTs may be involved in the regulation of genes with specific roles in normal or cancer cells. For instance, there are 6 homologues of H3K4 methyltransferases, denoted KMT2A to KMT2E, that are involved in methylation at this position. Moreover, one recent study has shown that KMT2A and KMT2B control different genomic regions in brain cells to regulate memory function^[25]^. Therefore, KMTs may serve as potential biomarkers in patients for individualized treatment. Depending on the lysine position, methylation state, and amino acids environment, histone lysine methylation can activate or repress gene transcription. Generally, methylation on H3K4, H3K36, and H3K79 are considered to activate gene transcription, while methylation on H3K9, H3K27 and H4K20 are thought to repress gene transcription^[8]^. In contrast to KMTs, one KDM can catalyse demethylation on several lysine residues. For instance, LSD1 (also called KDM1A) is specific to H3K4 and H3K9 residues^[26]^.

#### Short-chain lysine acylation

**Figure 1:**
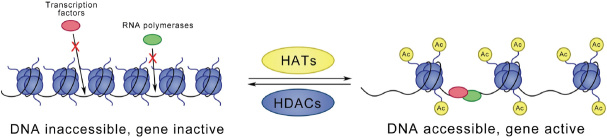
Acetylation or deacetylation of histone lysine residues is catalysed by HATs and HDACs, respectively. Lysine acetylation is connected to loosening of the chromatin structure. This change enables DNA binding and eventually leads to activation of gene transcription. In contrast, deacetylation closes the chromatin structure and represses gene transcription. HATs: histone acetyltransferases; HDACs: histone deacetylases

A classically studied lysine modification is acetylation of histone lysine residues. In a lysine acetylation reaction, an acetyl group from acetylated coenzyme A is transferred to the e-amino from a lysine residue, which results in neutralization of the positive charge and thus weakening of the electrostatic interaction with the DNA. This change leads to a more open chromatin structure, which allows access of DNA binding proteins. In general, acetylation is related to increased gene transcription, while deacetylation is connected to repression of gene transcription [Figure 1]. This dynamic process is catalysed by three groups of enzymes: (1) histone acetyltransferases (HATs), also known as “writers”, are responsible for transferring acetyl groups to targeted lysine residues; (2) histone deacetylases (HDACs), known as “erasers”, are found to remove acetyl groups; and (3) bromodomain proteins, known as “readers”, specifically recognize acetylated lysine residues.

Besides histone lysine acetylation, recent studies show that other short-chain CoAs, such as propionyl-CoA, butyryl-CoA^[27]^, 2-hydroxyisobutyryl-CoA^[13]^, crotonyl-CoA^[16]^, malonyl-CoA and succinyl-CoA^[28]^, can be used as substrates to acylate histone lysine residues.

### Others

Besides the aforementioned methylation and acetylation, other types of post-translational modifications are identified on histones, such as lysine ubiquitinoylation, sumoylation and ADP-ribosylation. These modifications are mostly reported to relate to DNA damage and repair. Moreover, phosphorylation of histone serine and threonine residues is a globally found modification, which plays important roles in diverse nuclear processes. Details for these modifications are discussed in recent reviews^[29-31]^.

## Aberrant histone modifications in cancers and the development of small-molecule inhibitors

### Inhibitors to target arginine modifications

Overexpression of PRMTs has been observed in various types of human cancers^[32]^. For instance, the overexpression of PRMT5 has been observed in non-Hodgkin lymphoma^[33,34]^. Additionally, recent studies show that PRMT5 promotes survival of lymphoma cells via WNT and AKT-mediated proliferation signalling^[35,36]^. Interfering with PRMT5 activity prevents the maintenance of malignant phenotypes^[37]^. Therefore, PRMT5 is a rational target for treating lymphoma. Small-molecule inhibitors specifically targeting PRMT5 have been developed and two inhibitors, JNJ-64619178 and GSK3326595, were patented and are now under clinical investigation. Besides the development of PRMT5 inhibitors, type I PRMT inhibitors have also gained interest due to the high expression of type I PRMTs in various types of cancers^[38-41]^. Moreover, PRMT1 is identified as an essential component of mixed lineage leukaemia (MLL) and specific knockdown of PRMT1 suppresses MLL-mediated transformation^[42]^. Interestingly, a recent study shows that GSK3368715, a type I PRMT inhibitor, synergizes with the anti-tumour effect of PRMT5 inhibition^[43]^ [Table 2].

**Table 2:** Inhibitors of histone methylation in clinical studies

Name	Type of histone modification	Target	Clinical phase	Condition or disease in clinic	Disease in preclinical studies
Pinometostat (EPZ-5676)	Lysine methylation	DOT1L	1	Advanced acute leukemia, particularly MLL-r^[44]^	Rearranged mixed lineage leukemia (MLL-r)^[45-47]^
CPI-1205		EZH2	1	B-cell lymphomas^[48]^	B-cell lymphomas^[49]^
Tazemetostat (EPZ-6438)			2	Elapsed or refractory B-cell non-Hodgkin lymphoma and advanced solid tumours^[50]^	Non-Hodgkin lymphoma^[51,52]^ Rhabdoid tumour models^[53]^
GSK2879552		LSD1	1	Relapsed or refractory SCL^C[54]^	Small cell lung carcinoma^[55]^
JNJ-64619178	Arginine methylation	PRMT5	1	Relapsed/refractory B cell non-Hodgkin lymphoma (NHL) or advanced solid tumours	Human NSCLC and SCLC cancer mouse xenograft models^[49]^
GSK3326595 (EPZ015938)		PRMT5	1	Advanced or metastatic solid tumours and non-Hodgkin’s lymphoma^[56,57]^	Hematologic and solid tumour cells lines^[58]^
GSK3368715 (EPZ019997)		Type I PRMTs	1	Solid tumours and diffuse large B-cell lymphoma	Lymphoma and AML cell lines^[43]^

SCLC: small cell lung cancer; NSCLC: non-small-cell lung carcinoma; AML: acute myeloid leukemia

### Inhibitors to target lysine modifications

Numerous studies have shown that mutation, dysregulation, or overexpression of lysine modifying enzymes such as KMTs, KDMs, HATs, or HDACs are associated with cancers and other diseases. Therefore, these enzymes were recognized as potential drug targets for cancer treatment^[59,60]^.

As listed in Table 2, several inhibitors targeting lysine methylation have been described. EZH2 (enhancer of zeste) homolog is becoming a potential target for treating lymphoma. EZH2 is a catalytic components of polycomb repressive complexes 2 (PRC2), which methylate H3K27^[61]^. Gain-of-function mutations of EZH2 are mainly detected in diffuse large B cell lymphoma and follicular lymphoma among all categories of lymphomas and lymphoid leukaemia^[62]^. Moreover, a mutation at Y641 within the catalytic domain of EZH2 proved to increase methylation of H3K27, because the mutant EZH2 shows higher catalytic efficiency compared to wild type EZH2. This increased methylation contributes to the pathogenesis of germinal centre B-cell lymphomas^[63]^. Another EZH2 mutation, A677G, also increases methylation of H3K27me3^[64]^. These insights triggered the development of EZH2 inhibitors for therapeutic use. For instance, tazemetostat is a promising inhibitor that is under investigation in phase II clinical trials.

Previously, the FDA approved several pan-HDAC inhibitors for the treatment of cancers. For instance, vorinostat (SAHA) is approved for the treatment of cutaneous manifestations of cutaneous T-cell lymphoma^[65]^. Belinostat (Beleodaq) is approved for the treatment of patients with relapsed or refractory peripheral T-cell lymphoma (PTCL)^[66]^, and panobinostat (Farydak) is approved for patients with relapsed multiple myeloma (MM)^[67]^. Besides these pan-HDAC inhibitors, a class I specific HDAC inhibitor romidepsin (Isodax) is approved for the treatment of PTCL. Further developments are aimed at the application of more isoenzyme-selective HDAC inhibitors. Moreover, several specific inhibitors are shown that were developed for cancer treatment over the last decade. Among these inhibitors, HDAC6-selective inhibitors show a promising anti-tumour effect to various cancers. For instance, ricolinostat (ACY-1215) shows strong potential at treating MM alone or with other drugs^[68-70]^. Several clinical trials using ricolinostat for patients with MM are currently ongoing (NCT01323751, NCT02189343, NCT01997840, and NCT01583283) [see Table 3].

**Table 3:** Specific HAT and HDAC inhibitors developed between 2009 and 2019, and their applications in cancer *in vitro*

Name	Target	Links to cancer
BG45	Class I HDAC	multiple myeloma^[71-73]^
TMP-195	Class IIa HDAC	Breast tumour^[74]^
LMK235	HDAC4,5	Chemoresistant cancer cells^[75]^ multiple myeloma^[76]^ pancreatic neuroendocrine tumours^[77]^
Tubastatin A	HDAC6	cholangiocarcinoma^[78]^ melanoma^[79]^
Ricolinostat (ACY-1215)		multiple myeloma^[68-70]^
SKLB-23bb		solid and hematologic tumour^[80]^
Cay 10603		Burkitt’s lymphoma^[81]^ lung carcinoma^[82]^
Nexturastat A		myeloma^[83-85]^
PCI-34051	HDAC8	neuroblastoma^[86]^ T-cell lymphomas^[87]^ malignant peripheral nerve sheath tumours^[88]^
A485	P300/CBP	myeloma^[89,90]^

HAT: histone acetyltransferase; HDAC: histone deacetylase

In comparison to HDAC inhibitors, the development of potent and specific HAT inhibitors is lagging. C646 was firstly considered as a p300 and CBP selective inhibitor^[91]^. However, a recent study shows that C646 binds off-target to other kinases^[92]^. A novel p300 and CBP specific inhibitor A485 was synthesized and shows inhibition of proliferation in myeloma cells^[90,93]^. This new inhibitor holds promise for further exploration in myeloma.

Bromodomains are protein modules that are present in 46 different human proteins^[94]^. An important bromodomain family is the bromodomain and extra-terminal domain-containing (BET) protein family, which consists of two bromodomains (BD1 and BD2) and one extra-terminal domain. BET proteins recognize and bind specific peptide sequence in the chromatin, which plays an enabling role in the assembly of protein-protein complexes on the chromatin^[95]^. Currently, BET protein modules are considered as a promising group of targets for treatment of cancer, which triggered the development of BET inhibitors^[96-98]^. Whereas early studies provided BET inhibitors with limited selectivity, more recent studies promise to provide therapeutically relevant BET inhibition with improved selectivity profiles. For instance, ABBV-744 and BY27 are BD2-specific inhibitors that are proven to inhibit tumour cell growth *in vitro*^[99,100]^. A phase I study is ongoing in which the safety and pharmacokinetics of BET inhibitor ABBV-744 is evaluated for treatment of patients with acute myeloid leukaemia (NCT03360006).

## Improved trail-induced apoptosis by targeting enzymes involved in histone modifications

### TRAIL-induced apoptosis pathways

**Figure 2:**
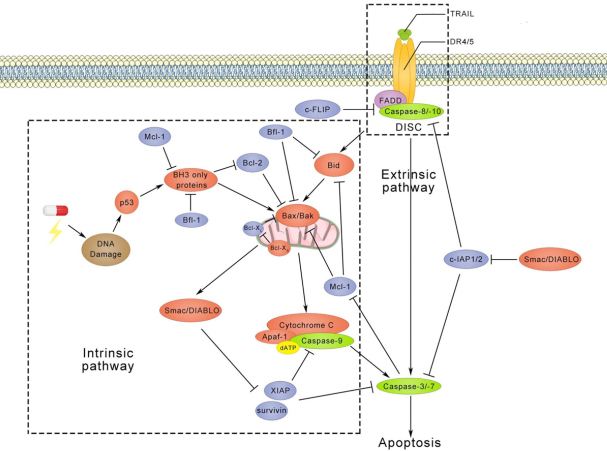
TRAIL-induced apoptotic pathways. After trimerization, TRAIL binds to death receptors, which triggers the formation of the DISC and activates caspase-8/10. Subsequently, activated caspase-8/10 induces cleavage of caspase-3/7, which leads to apoptosis. On the other hand, cleaved caspase-8/10 can also recruit Bid to trigger apoptosis via the intrinsic pathways. The intrinsic pathway is usually activated by DNA damage followed by p53 activation, whereas TRAIL-induced intrinsic apoptotic pathway is independent of p53. Interestingly, p53 has also been found to regulate TRAIL receptors DR4, DR5, DcR1, and DcR2^[104-107]^. Anti-apoptotic proteins, including c-FLIP, c-IAP1/2, Bcl-2, Bfl-1, Mcl-1, Bcl-X_L_, XIAP, and survivin, are shown in blue circles. DISC: death-inducing signalling complex; DR4: death receptor 4; DR4: death receptor 5; DcR1: decoy receptor 1; DcR2: decoy receptor 2

TRAIL is a member of the TNF superfamily and it binds to five receptors, including death receptor 4 (DR4), death receptor 5 (DR5), decoy receptor 1 (DcR1), decoy receptor 2 (DcR2), and osteoprotegerin. DR4 and DR5 both contain an intracellular death domain (DD), which initiates apoptotic signalling transduction. In contrast, DcR1 and DcR2 do not induce apoptosis due to the truncated DD in DcR1 and the absent DD in DcR2. The mechanisms of TRAIL-induced apoptosis have been intensively investigated and pathways identified are shown in Figure 2^[101-103]^. Extrinsic apoptotic signalling is initiated upon binding of a TRAIL trimer to DR4 or DR5, which initiates formation of a death-inducing signalling complex (DISC). In this DISC, FAS-associated protein with death domain (FADD) is connected with DR4 or DR5 via DDs. Initiator caspases, like pro-caspase-8 or 10, are recruited to FADD via the interaction between death effector domains. This recruitment also actives self-dimerization of pro-caspase-8 or 10, leading to auto-proteolytic processing at consensus cleavage sites. Executioner caspases, like caspase-3 or 7, are cleaved by initiator caspases to create a mature functional protease, which coordinates to the degradation phase of apoptosis, including DNA fragmentation, membrane blebbing and cell shrinkage. Single active executioner caspase can cleave and activate other caspases, resulting in activation of the caspase cascade. In addition, caspase-8 or 10 engages the intrinsic apoptosis pathway through cleavage of the BH3-interacting domain death agonist (Bid) to facilitate the release of cytochrome C from mitochondria. In fact, the truncated Bid translocates from the cytoplasm to mitochondria and stimulates oligomerization of Bax or Bak. At the same time, Bax and Bak permeabilize the membrane of the mitochondrion, also called mitochondrial outer membrane permeabilization (MOMP). Following MOMP, the mitochondrial inner membrane releases cytochrome C or second mitochondria-derived activator of caspase/direct inhibitor of apoptosis-binding protein with low pI (Smac/DIABLO) into the cytosol. With the binding of cytochrome C to adaptor protein apoptotic protease-activating factor-1, dATP and the initiator caspase caspase-9 are recruited to form the apoptosome. Finally, active caspase-9 directly cleaves executioner caspases caspase-3 or 7.

Anti-apoptotic proteins are also involved in these apoptotic signalling pathways. For instance, cellular-FLIP (c-FLIP) and cellular inhibitors of apoptotic proteins (cIAP1 and cIAP2) disturb the formation of DISC. X-linked IAP (XIAP) and survivin, on the other hand, block executioner caspases and the apoptosome. Moreover, anti-apoptotic Bcl-2 family members, like Bcl-2, Bcl-X_L_, Mcl-1, and Bfl-1 are able to prevent MOMP.

### Improving TRAIL-induced apoptosis

Although, TRAIL has promising tumour-cell selective apoptosis-inducing properties, various tumour cells are resistant to TRAIL treatment. Therefore, it is important to improve TRAIL-sensitivity. Here, we discuss the strategies of improving TRAIL-sensitivity by targeting histone modifying enzymes that are involved in methylation and acetylation. Examples of the use of selective inhibitors as TRAIL sensitizers to overcome TRAIL-resistance are shown in Table 4.

**Table 4:** Improved TRAIL-induced apoptosis pathway using inhibitors targeting enzymes in histone modifications

Target	Small molecule	Regulation mechanisms	Cancer type	Ref.
Euchromatic histone-lysine *N*-methyltransferase 2 (EHMT2, G9a)	BIX-01294	Downregulation of Survivin and Upregulation of DR5	Renal carcinoma	[108]
		Upregulation of DR5	Breast cancer	[109]
PRC2	Retinoic acid (RA) or 3-deazaneplanocin A (DZNep)	Increased DR5 transcript level	Colon cancer	[110]
Class I HDAC	Entinostat (MS-275)	Restore expression of Coxsackie Adenovirus receptor	Prostate cancer	[111]
		Upregulation of DR4, DR5, Bax, Bak	Breast cancer	[112]
		Decrease degradation of endogenous TRAIL	Anaplastic thyroid carcinoma	[113]
		Expression of endogenous TRAIL	Acute myeloid leukemia	[114]
HDAC3	RGFP966	Upregulation of DR4	Colon cancer	[115]
HDAC8	PCI34051			

#### Histone methylation

The enzyme EHMT2 catalyses the dimethylation of H3K9me2, which is associated with silencing of tumour suppressor genes. The PRC2 complex plays an important role in H3K27me3, which is also related to transcriptional repression of tumour suppressor genes. When combined with TRAIL, inhibitors of either EHMT2 or PRC2 increase the number of apoptotic cells through upregulation of DR5^[108,109]^. These results indicate that the expression of DR5 may be be related to the reduced methylation of histones.

Additionally, a recent study shows that silencing KDM2B, a H3K36-specific histone demethylase, can cause a de-repression of a pro-apoptotic gene Harakiri (HRK) in glioblastoma multiforme cells. This study also shows that the silencing of KDM2B cooperates with TRAIL to reduce cell viability^[116]^.

As discussed above, EZH2 is a promising therapeutic target for lymphoma. Therefore, EZH2-specific inhibitors may enhance the sensitivity of lymphoma cells to TRAIL. Additionally, another methyltransferase PRMT5 has been identified as a novel TRAIL receptor binding protein at the plasma membrane, which is involved in the early stage of signal initiation for induction of the NF-κB signalling pathways^[117]^. Moreover, a study shows that the overexpression of PRMT5 increased expression of c-FLIP_L_ by decreasing the ubiquitination via inhibition of the interaction between c-FLIP_L_ and ITCH, leading to decreased apoptotic cells induced by doxorubicin in human lung cancer cells^[118]^. Therefore, targeting PRMT5 by specific inhibitors may improve sensitivity to TRAIL.

#### Histone acetylation

Previously, studies have shown that the combination of pan-HDAC inhibitors, such as panobinostat, with TRAIL downregulates anti-apoptotic proteins, c-FLIP and XIAP, thereby improving sensitivity to TRAIL^[119,120]^. This study indicates a close relationship of histone acetylation and the TRAIL signalling pathways.

Moreover, highly acetylated Ku70, a DNA repair protein, disrupts the formation of the Ku70-FLIP complex and triggers the degradation of FLIP by polyubiquitination. Therefore, using the HDAC inhibitor vorinostat increases apoptosis through the stabilization of the Ku70-FLIP complex in colon cancer models *in vivo*. Interestingly, this study also shows that the HDAC6-specific inhibitor tubacin increases apoptosis^[121]^. With the increasing development of HDAC-specific inhibitors, the combination of HDAC specific inhibitors with TRAIL may be an interesting choice [Table 4].

Interestingly, the BET inhibitor JQ1 was reported to reduce the expression of c-FLIP and XIAP at mRNA and protein level in KRAS-mutated NSCLC cells. Combined JQ1 with TRAIL significantly enhanced apoptosis^[122]^. Furthermore, JO1 combined with the HDAC inhibitor vorinostat increases apoptosis via the extrinsic pathway in CTLC cells^[123]^. These results indicate that BET inhibitors play an important role in regulating proteins in the apoptotic signalling pathway axis. Therefore, the combination of BET inhibitor with TRAIL may be a promising strategy for the development of cancer therapeutics.

## Conclusion

Due to intensive research efforts over the past decades, the knowledge of epigenetic regulation in carcinogenesis is expanding rapidly. This knowledge provides new insights into the role of histone modifications in oncogenic gene transcription. Consequently, histone modifying enzymes have been recognized as drug targets. In this review, we summarize recent discoveries involving histone modifications and the enzymes involved. We focus on small molecules targeting these enzymes involved, and we highlight their effects on TRAIL-induced apoptosis. Finally, we indicate new targets in Table 4 for enhancing TRAIL sensitivity.

## Declarations

### Copyright

© The Author(s) 2020.
